# Internet Interventions for Long-Term Conditions: Patient and Caregiver Quality Criteria

**DOI:** 10.2196/jmir.8.3.e13

**Published:** 2006-07-28

**Authors:** Cicely Kerr, Elizabeth Murray, Fiona Stevenson, Charles Gore, Irwin Nazareth

**Affiliations:** ^2^The Hepatitis C TrustLondonUnited Kingdom; ^1^Department of Primary Care and Population SciencesLondonUnited Kingdom

**Keywords:** Internet, patients, qualitative research, interactive health communication applications

## Abstract

**Background:**

Interactive health communication applications (IHCAs) that combine high-quality health information with interactive components, such as self-assessment tools, behavior change support, peer support, or decision support, are likely to benefit people with long-term conditions. IHCAs are now largely Web-based and are becoming known as "Internet interventions." Although there are numerous professionally generated criteria to assess health-related websites, to date there has been scant exploration of patient-generated assessment criteria even though patients and professionals use different criteria for assessing the quality of traditional sources of health information.

**Objective:**

We aimed to determine patients' and caregivers' requirements of IHCAs for long-term conditions as well as their criteria for assessing the quality of different programs.

**Methods:**

This was a qualitative study with focus groups. Patients and caregivers managing long-term conditions used three (predominantly Web-based) IHCAs relevant to their condition and subsequently discussed the strengths and weaknesses of the different IHCAs in focus groups. Participants in any one focus group all shared the same long-term condition and viewed the same three IHCAs. Patient and caregiver criteria for IHCAs emerged from the data.

**Results:**

There were 40 patients and caregivers who participated in 10 focus groups. Participants welcomed the potential of Internet interventions but felt that many were not achieving their full potential. Participants generated detailed and specific quality criteria relating to information content, presentation, interactivity, and trustworthiness, which can be used by developers and purchasers of Internet interventions.

**Conclusions:**

The user-generated quality criteria reported in this paper should help developers and purchasers provide Internet interventions that better meet user needs.

## Introduction

One aspect of eHealth is patients' use of new technologies to become better informed about their health and health care options [1].

In response to consumers' desire for information that enables them to play an active role in their health care, there has been a proliferation of health-related websites on the Internet. The interactive nature of the Internet, combined with the potential to store large volumes of information, provides a unique opportunity to offer high-quality interactive evidence-based information. Interactive components such as self-assessment tools permit the provision of personalized tailored information to users and provide decision support, peer support, or behavior change support. This combination of health information and interactive components is known as an interactive health communication application (IHCA) [2]. Initially, IHCAs were often developed on non-Web-based platforms such as CD-ROM. Recently, the emphasis has moved toward Web-based programs, which are becoming known as Internet interventions [3].

This combination of high-quality health information with interactive components is likely to benefit people with long-term conditions [4,5] who can be conceptualized as being on an "illness journey" [6]. As they progress through their journey, they experience changing needs [7], often becoming highly knowledgeable about their health problems and developing substantial expertise in self-management [8]. In a recent systematic review of IHCAs for chronic conditions, IHCAs were found to have largely positive effects, tending to improve user knowledge and perceived social support. The review also suggested that these positive effects of IHCAs may extend to improved clinical outcomes [2]. However, there has also been anxiety about the potential harms caused by health-related Web-sites, particularly when the information provided is misleading or incorrect [9].

One response to these concerns has been the development of criteria to assess the quality of health-related websites. Numerous such criteria, mostly generated by professionals, have been proposed [10-12]. The criteria tend to reflect professional concerns, including accuracy, completeness, readability, disclosures, and references [11]. By contrast, little is known about the user perspective on health websites; however, we know that patients and professionals generate different criteria for assessing the quality of traditional non-Web-based information materials [13], suggesting that patients are likely to use different criteria than professionals for assessing the quality of health websites. In a qualitative study, Eysenbach observed healthy volunteers to determine how they found and appraised the quality of health websites [14]. The Pew Internet and American Life Project undertook a large questionnaire study of Internet users to determine how respondents appraised the quality of health websites [15]. However, people with long-term conditions have different information and health needs to healthy volunteers and hence may use different criteria for assessing the quality of interactive health websites.

A further limitation of most quality criteria and previous user perspective research is that they do not distinguish between sites which contain information only and interactive sites which combine information with decision support, behavior change support, or peer support. This distinction is important as information on its own is relatively ineffective in achieving behavior change or improving clinical outcomes [16]. While steps have been made to develop criteria to evaluate more interactive online health behavior change and disease management programs [17], these also neglect the user perspective.

We aimed to determine the criteria used by people with long-term conditions and their caregivers for assessing the quality of IHCAs (or Internet interventions). As we were interested in user-generated criteria, we opted for a qualitative rather than a quantitative methodology (such as a questionnaire study that would have forced participants to choose between predefined criteria generated by the researcher).

## Methods

Patients and caregivers managing long-term conditions used three IHCAs relevant to their condition and subsequently discussed the strengths and weaknesses of the different programs in focus groups. Participants in any one focus group all shared the same long-term condition and viewed the same three IHCAs. Patient and caregiver criteria for IHCA emerged from the data.

### Sample

We selected a range of long-term conditions to cover: conditions that are highly prevalent in the UK population and account for substantial morbidity or mortality; conditions in which self-care is known to, or likely to, affect clinical status; and conditions that affect different age groups, including children, adults, and older adults. In addition, we aimed to include a highly stigmatized condition and one for which the scientific evidence base for treatment is changing rapidly (patients with such conditions may have particular need of an IHCA).

We recruited adult patients with diabetes mellitus, ischemic heart disease, or hepatitis C, parents of children with asthma or diabetes mellitus, and caregivers of people with Alzheimer's disease.

### Recruitment Strategies

In order to recruit a maximally diverse sample, we used a range of recruitment strategies, including recruiting from both clinical and community settings in three UK areas with differing socioeconomic and ethnic profiles. These were inner London (urban, very mixed ethnically and socioeconomically); Nottingham (medium-sized city, mostly lower socioeconomic status, high proportion of South Asian residents); and Exeter (a small city set in a rural area, mostly indigenous English residents). People were invited to take part through advertisements in local newspapers and patient newsletters, posters in general practice (family practice) clinics, and flyers given out in patient self-help group meetings, exercise classes, and hospital outpatient clinics. Recruitment continued until we had sampled to the point of redundancy (ie, until no new data were emerging from the focus groups).

### Intervention

Suitable IHCAs were identified through authors of studies reporting on the development and/or evaluation of IHCAs in the academic literature, Google Internet searches for each of the relevant long-term conditions, and by asking researchers, academics, and consumer representatives for interventions known to them personally. We excluded programs that only provided health information without any interactive components, as these do not meet the definition of an IHCA, and those that were aimed at more than one condition. We wanted to show participants three programs that differed significantly, so we compiled a list of programs developed by different stakeholders (medical, academic, commercial, charitable, and self-help organizations) from different countries (although all using the English language). Table 1 provides details of the interventions selected. Although we planned to use IHCAs on any available technological platform, all but one was Web-based, and hence we use the simpler more intuitive term of Internet interventions in reporting and discussing our results.

**Table 1:** Details of IHCAs shown to participants

**Condition**	**IHCA**	**Details**
Adult patients with diabetes	-My Diabetes -Freely available at www.mydiabetes.com	-Identified by Google Internet search -Produced by US commercial stakeholder
	-Diabetes Insight -Freely available at www.diabetes-insight.info	-Identified by Google Internet search -Produced by UK charitable stakeholder
	-Aida -Freely available at www.2aida.org	-Identified via systematic review search of academic literature -Produced by European academic stakeholders
Adult patients with heart disease	-Heart center online -Freely available at www.heartcenteronline.com	-Identified by Google Internet search -Produced by US commercial stakeholder
	-Your Heart -Freely available at www.yourheart.org.uk	-Identified by Google Internet search -Produced by UK National Health Service (NHS) and academic stakeholders
	-Heart info -Freely available at www.heartinfo.org	-Identified by Google Internet search -Produced by US charitable stakeholder
Adult patients with hepatitis C	-Hepatitis C forum -Freely available at www.hepatitis-c.de	-Identified by consumer representative -Produced by German forum (stakeholder unclear)
	-Hep C vets -Freely available at www.hepcvets.com	-Identified by consumer representative -Produced by a US consumer representative stakeholder
	-Hep C UK -Freely available at www.hepCuk.info	-Identified by consumer representative -Produced by UK charitable consumer representative stakeholder
Caregivers of people with Alzheimer's disease	-Alzheimer's disease -Freely available at www.alzheimersdisease.com	-Identified by Google Internet search -Produced by US commercial stakeholder
	-Alzheimer Society -Freely available at www.alzheimers.org.uk	-Identified by Google Internet search -Produced by UK charitable consumer representative stakeholder
	-CHESS AD -Web-based: permission, access passwords, and log-in provided by CHESS project, University of Wisconsin	-Identified via systematic review search of academic literature -Produced by US academic stakeholder
Parents of children with diabetes	-Juvenile diabetes research foundation -Freely available at www.jdf.org	-Identified by Google Internet search -Produced by US charitable stakeholder
	-American Diabetes Association -Freely available at www.diabetes.org/for-parents-and-kids.jsp	-Identified by Google Internet search -Produced by US charitable stakeholder
	-Children with diabetes -Freely available at www.childrenwithdiabetes.com	-Identified by Google Internet search -Produced by US commercial stakeholder
Parents of children with asthma	-National asthma campaign -Freely available at www.asthma.org.uk	Identified by Google Internet search Produced by UK charitable stakeholder
	-CHESS asthma -Web-based: permission, access passwords, and log-in provided by CHESS project, University of Wisconsin	-Identified via systematic review search of academic literature -Produced by US academic stakeholder
	-The Asthma Files -CD-ROM: permission provided by Dr Alan Smyth, Nottingham City Hospital	-Identified via systematic review search of academic literature -Produced by UK medical stakeholders

### Focus Groups

All the participants of any one focus group had the same long-term condition. Group size ranged from 2 to 8 participants. Groups were run at community information technology (IT) facilities to avoid a health service context. Participants initially accessed a networked personal computer (PC), pre-loaded with the three interventions. Individual participants used each Internet intervention for up to 30 minutes, spending up to 90 minutes at the PC. During this time, participants were free to explore each IHCA as they wished, to form an opinion of how useful it might be to them and be able to discuss this later. Facilitators assisted novice computer users, and participants were encouraged to make contemporaneous notes on the three IHCAs to serve as a memory aid for subsequent discussion.

After viewing the three IHCAs, participants re-convened for a 90-minute discussion facilitated by two experienced focus group facilitators. One facilitator led the discussion and the other served as an observer, making contemporaneous field notes. The discussions were tape-recorded.

### Topic Guide

The topic guide was developed following a review of the literature and discussion with relevant researchers (Multimedia Appendix). Minor modifications were made following piloting with user representatives of hepatitis C and diabetes patients. Data emerging from some of the early groups influenced follow-up and probe questions with later groups, but without altering the topic areas. Areas covered in the topic guide included participants' overall reactions to the three IHCAs, preferences for and against particular IHCAs (and reasons for these), and information looked for but not found.

### Analysis

Audiotapes of the discussions were transcribed verbatim. Each participant was given a unique anonymous identifier based on focus group location, disease condition, and chronological order of focus group. Hence, EHD refers to a participant from Exeter with heart disease, while LCD is a participant from London who cares for a child with diabetes. For clarity, each quote is also labeled with the focus group number and condition shared by participants. Analysis was conducted on un-edited transcripts, but for clarity, edited quotes are presented in the results section.

Analysis and data collection were conducted concurrently, starting as soon as the first audiotape had been transcribed. Thematic analysis of each transcript identified emerging requirements and quality criteria. Analysis was organized into an expanding list of themes and subthemes, assisted by using QSR NUD*1ST 6 software [18]. Analysis conducted initially by one researcher was checked for validity against observational research notes and discussed with the two focus group co-facilitators. The iterative process of data collection and analysis served to inform discussions in later groups. Follow-up and probe questions explored agreement or disagreement, with views expressed by earlier groups providing further detail and clarification of emerging themes. Focus groups with patients were conducted until the point of saturation, when no new themes were emerging. Focus groups with caregivers were curtailed by lack of caregiver participation.

The list of emerging themes was discussed with members of the multidisciplinary research team (representing clinical, health psychology, sociology, and consumer perspectives) before being summarized into a framework. All utterances expressing a judgment in the transcripts were then coded using this framework.

### Respondent Validation

Once the initial analysis was complete, the resulting criteria were sent to focus group participants and those who had consented to participate in the study but had not been able to attend a focus group. Respondents were asked to rate each criterion on a scale of 1 (not at all important) to 5 (essential) and select the three criteria that they felt were most important. There was also space for respondents to add any criteria or topics that they felt were missing from the list.

## Results

### Sample

A total of 40 patients and caregivers participated in focus groups. An additional 40 people consented to participate in the study but were unable to attend focus groups or attended when no others did. Finding time to attend a focus group was particularly problematic for caregivers (including parents) who had other demands on their time. Focus group attendees consisted of roughly equal numbers of men and women. The sample was diverse in terms of age, ethnic background, previous computer literacy, and educational background (Table 2). Nearly one quarter of participants had left school at age 16 (the minimum allowable age in the United Kingdom), and nearly one third had either no, or only basic, previous computer experience.

**Table 2:** Self-reported characteristics of participants (N = 40)

**Number of Participants (%)**		
Gender	Male	22 (55)
	Female	18 (45)
Age	30-39	3 (7.5)
	40-49	7 (17.5)
	50-59	8 (20)
	60-69	16 (40)
	70-79	4 (10)
	Missing data	2 (5)
Employment	Employed	12 (30)
	Economically inactive	27 (67.5)
	Missing data	1 (2.5)
Education	School leaver	9 (22.5)
	A levels or vocational equivalent (A levels are advanced level examinations taken at age 18)	9 (22.5)
	University degree, Higher National Diploma, or similar	20 (50)
	Missing data	2 (5)
Ethnic group	White British	31 (77.5)
	White European (non-British origin)	6 (15)
	Asian or British Asian	2 (5)
	Black or Black British	1 (2.5)
English first language	Yes	34 (85)
	No	6 (15)
Computer experience	Novice	4 (10)
	Basic	8 (20)
	Experienced	28 (70)
Time since diagnosis	Less than 1 year	4 (10)
	Between 1 and 5 years	12 (30)
	More than 5 years	18 (45)
	Missing data	6 (15)

### Focus Groups

We ran 10 focus groups, each attended by 2 to 8 participants. Focus groups were run for all conditions except for parents of children with asthma, who were not able to attend at the same time (Table 3).

### Response to Internet Interventions

Overall, participants highly valued Internet interventions. They welcomed the existence of these programs, and were highly appreciative of their potential:

LAD20; G2, Diabetes "Totally unbounded potential, the potential to step in and alleviate lots of conditions."

However, it was clear that participants felt many of the interventions were not fulfilling their potential. Participants could see strengths and weaknesses of the various programs they explored, and they generated criteria that were generic across patient and caregiver groups that related to information content, presentation, interactive components, and trustworthiness.

**Table 3:** Summary of focus groups by condition

**Condition**	**Number of Groups**	**Number of Participants**	**Locations**
Adult patients with diabetes	4	13 (3 with comorbid heart disease)	London and Nottingham
Adult patients with heart disease	3	17 (2 with comorbid diabetes)	London and Exeter
Adult patients with hepatitis C	1	4	London
Caregivers of people with Alzheimer's	1	3	London
Parents of children with diabetes	1	3	London
Total	10	40	

### Information Content

Participants tended to see the information content as the single most important feature of an Internet intervention and hence generated more criteria relating to information content than to presentation, trustworthiness, or interactive components. Information content criteria apply to all the information in the intervention, including that provided in the interactive components. Criteria relating to information content are presented in Textbox 1.

#### Evolving Information Needs

Participants recognized that one of the potential strengths of Web-based information was the ability to provide an almost unlimited volume of information, and they wanted this reflected in the level of detail provided. Participants stated that information needs evolve as patients and caregivers become more experienced with managing their condition, and that a good Internet intervention should address the needs of both newly diagnosed patients and people who are already knowledgeable about their condition. Internet interventions were frequently criticized for providing basic information only. More knowledgeable users wanted access to in-depth scientific information about the condition and specific treatments, with many wanting information about new research and promising future treatments. They wanted Internet interventions to contain detailed, specific, and practical information covering the wide range of topics in Textbox 1.

LCD07; G8, Parent of diabetic child "I think it's quite easy to find background information…it's the sort of reviewing things and revisiting and reassessing and keeping your eye on the ball…that's missing."

LHD09; G6, Heart disease "You actually accumulate quite a lot of information…on the way and so we're probably asking for more specific things and quite a lot of information."

**Patients' and caregivers' quality criteria for Internet interventions relating to information content**

**Information content:**

- Content needs to be detailed, specific, and of practical use.
- Long-term use requires increasing depth of information as self-management experience grows, as well as new and up-to-date information.

**A good Internet intervention will provide information about the following:**

- What to expect of the condition and treatment (eg, usual course of the condition, possible complications, tests and treatments that may be offered).
- Medication (eg, indications for use and potential side effects).
- Available treatments in the United Kingdom and elsewhere.
- In-depth scientific information about the condition and treatments.
- The practicalities of day-to-day living (eg, going on holiday, traveling, planning what food to buy and eat).
- Practical information (eg, guidance on what relevant books and gadgets are available and where to buy them, information about legal issues and benefits available, including completed examples of relevant forms, letters, and templates).
- Local services and resources (eg, local health services, voluntary organizations, and self-help groups).
- New research and areas of scientific or medical uncertainty (eg, new research presented with an evaluation of the available evidence base and current practice).
- Conflicting expert or scientific views, with an explanation of what this uncertainty means for users (eg, new or emerging research or complementary therapies).
- Other people's experiences (eg, personal stories from other people with similar health problems, other people's questions and answers, facility to interact with other people).
- Information for family members, addressing the concerns and roles of those around them.

**Other criteria particularly related to information content criteria:**

**Manage the quantity and depth of information available.**

- Allow the user to control how much information, and on what topic, they access at any one time.
- Users need to easily access understandable information on the correct topic and to easily find the correct level for them.

**Ensure all information is accurate and up-to-date.**

- This means dating entries, providing information about the frequency and means of updating, and referring to recent media stories and developments.

#### Scientific Uncertainty

Participants held a range of views on how Internet interventions should deal with scientific uncertainty. While some participants wanted to access all the latest research results and decide on their validity for themselves, others were concerned that unproven or uncertain information would undermine more generally accepted advice. Some participants favored setting a threshold of scientific acceptability before reporting new findings, but it was unclear how this threshold would be determined. Others felt that it would be sufficient to provide an evaluation of the strength of new evidence, highlighting areas of uncertainty and reasons for treating initial findings with caution.

LHD07; G6, Heart disease "I'd rather know and know what the caveats are and what [the] sort of limitations of my access to it are…. I'd rather feel fully informed than not informed enough."

EHD05; G10, Heart disease "…confident that the research is sound, that it is peer reviewed…and…enough people have volunteered."

#### Practical Information

Users wanted practical information to help with the activities of daily living, such as shopping, meal planning, and exercising, as well as help with potentially difficult situations such as going on holiday or traveling. Users looked for information that would help friends and family plan activities. Other people's experiences, provided through personal stories, question and answer forums, or chat room facilities, were considered a particularly good source of practical information.

LAD04; G4, Diabetes "But it's actually the practical day-to-day living of it and your lifestyle management that you really need to be really clued up on."

LHD07; G6, Heart disease "Rather than reinventing the wheel, it's sharing with other people and there are hints and tips that you get from them that you just wouldn't get from a GP, just little practical things."

#### Managing Access to Information

Users wanted to be able to control the amount and detail of information they accessed and not be forced to see potentially upsetting or overly complex information when they did not feel ready for it.

LHD05; G6, Heart disease "We should have a choice of knowing…because all these websites presented you with information, whether you wanted to know it or not."

#### Updating Information

Participants were highly critical of sites that were not regularly updated, and they wanted entries dated to help users assess how frequently sites were updated.

LAD02; G4, Diabetes "The moment I saw the date 2000 it kind of shut me down, 'cos this is 2004…. I really wanna see end of 2003/2004."

### Presentation

#### Navigation

Participants stressed the need for easy navigation that allowed swift access to relevant information (Textbox 2). Sites that heavily relied on drop-down menus on the home page or contained the "back" button as the only way of exiting from a line of enquiry were criticized as being "frustrating" (LHD08; G6, Heart disease). Sites with multiple hyperlinks were praised as being "straightforward" (LAC04; G7, Alzheimer's caregiver), "idiot proof" (LHD10; G3, Heart disease), or "user friendly" (NAD02; G9, Diabetes).

#### Visual Appearance

The overall appearance of an Internet intervention contributed significantly to its appeal, for purely esthetic reasons, by enhancing usability, or by contributing to the tone of the information. Ideally, the site should strike a balance between being "too busy" (LCD09; G8, Parent of diabetic child) or too "tabloid" (LAD04, G4, Diabetes) on the one hand, and too "serious looking" (LCD09; G8, Parent of diabetic child) or too "bland" (LAD02; G4, Diabetes) on the other.

Patients and caregivers preferred sites where information was visually presented in various formats as "everybody learns differently" (LAD02; G4, Diabetes) and visual information helps you "see what actually happened" (LHD08; G6, Heart disease).

#### Language and Tone

Language and tone were considered very important. Participants universally disliked the use of unexplained medical jargon or non-UK terminology. However, use of technical or medical terms was considered necessary to convey information accurately, and also to help users communicate with their health care professionals, as long as the terms were defined and explained. Language and appearance combined to set the tone of an Internet intervention. While the wrong tone could be off-putting, the correct tone reassured users. Participants did not like overly "worthy" sites (LAD20; G2, Diabetes), but preferred a site to be "no-nonsense" (LHC02; G5, Hepatitis C), "non-patronizing" (LAD04; G4, Diabetes), and "authoritarian but friendly" (LHC01; G5, Hepatitis C).

Two elements linked presentation to other concerns: logging in and links to other sites.

#### Logging In

Participants did not like sites that required users to log in. Novice users found it hard to do, while others found it time consuming. Participants were put off by having to provide personal information before accessing content, particularly if it meant providing personal details or a user name. Users were concerned about the trustworthiness of a site that required them to log in, as they were concerned about the security of personal information, or as one participant put it, "Who has the back door key for it?"(LAD20; G2, Diabetes).

#### Links to Other Sites

Participants preferred Internet interventions that provided comprehensive and consistent information, and that did not continually send users "off-site." Many users found it difficult to return to the home site after following a link to another site. Participants stated that they wanted clear notification of being taken "off-site" so they could judge the trustworthiness of any external site.

**Patients' and caregivers' quality criteria for Internet interventions relating to presentation**

**Presentation:**

- The presentation needs to facilitate easy and speedy access to relevant information content.
- It needs to be attractive, engaging, understandable, and visually varied.

**A good Internet Intervention will have excellent Web design:**

- Easy navigation, including rapid and easy return to the home page; easy to locate search engines that search within the confines of the site and show intelligence by responding meaningfully to searches conducted using simple terms; use of hyperlinks to link up various sections of information within a site and for easy navigation by novice users; site maps for easy navigation by more experienced users.
- An attractive appearance, using colors, graphics, videos, animations, photos, and text broken up into small sections.
- Use of plain English, with a straightforward, but not patronizing tone; medical terms and jargon should be explained, but not avoided.

**Other criteria particularly related to presentation criteria:**

**Logging in**

- Not unnecessarily requiring users to log in or enter personal details before allowing access information.

**Links to other sites**

- Only for additional information and resources, with clear warnings about being taken off-site and summaries of information content and other relevant details of other sites.

### Interactive Components

The interactive features discussed in focus groups included personalized online assessments with personalized advice, Ask the Expert facilities, and online peer support groups. Participants had a range of views about the interactive components of the websites. Almost all felt that some degree of interactivity was helpful as it made the site more appealing and easier to use.

LACO1; G7, Alzheimer's caregiver "I enjoyed the fact it was more interactive…. I found it entertaining."

Some valued the specificity and tailoring of advice and information that could follow completion of online self-assessment tools, stating that this was the best way of obtaining personalized advice (short of seeing a doctor). These users were also keen on facilities such as "Ask the Expert," which allow users to put questions to specialist advisors.

LAD14; G2, Diabetes "Yes, it would appeal to you because you think Well they're looking at me specifically and they're guiding me.… So what is good for me, because I'll be different to you and to him."

However, others were concerned that they might inadvertently enter incorrect information and hence receive inappropriate or unsafe advice.

Many participants favored online peer support and electronic discussion groups, seeing them as a nonjudgmental source of support from people facing similar issues and challenges, available 24 hours a day.

LHC03; G5, Hepatitis C "I do think it's very helpful because 3 o'clock in the morning…I just felt like not wanting to go on any more. Where do I go to get help at 3 o'clock in the morning? There isn't anywhere, whereas if there's a website where you can go in and talk to somebody else that's going through the same, it might be helpful."

LAC03; G7, Alzheimer's caregiver "So you just write what you feel and hopefully somebody can give you something back without any risk of judgment."

However, others said that they were well supported already and could not see the need for such online groups.

The divergent views of participants account for the criteria generated in that they felt Internet interventions need to provide multiple interactive components that are optional, allowing the user to chose which, if any, interactive features to use (Textbox 3).

**Patients' and caregivers' quality criteria for Internet interventions relating to interactive components**

**Interactive components:**

- These contribute to the tone of Internet interventions.
- They need to provide multiple, optional, interactive components and allow users to choose which, if any, to use.

**A good Internet Intervention will include a range of interactive components:**

- Personalized online assessments, advice, and monitoring of the condition
- Online facility for asking an expert questions about the condition or treatment
- A question and answer facility or online chat room for online questioning and discussion with other people with similar health problems

### Trustworthiness

Trustworthiness was very important to participants, but they wanted to be able to evaluate a site's trustworthiness swiftly, based on what they already knew about the authors of the site. They did not want to have to look for credentials, disclaimers, or privacy policies of unfamiliar individuals or organizations. Some participants felt that a kite mark or quality seal from a recognized center would be helpful.

LAD20; G2, Diabetes "I want to go to a site where there's a seal…a stamp, as long as I knew what the stamp was."

The presence of adverts or commercial sponsors made users wary of the information provided.

LACO4; G7, Alzheimer's caregiver "They [drug companies] lie. They've got to skew the facts in their direction, that's what they are there for. They've got to sell their product."

Another feature that was important for developing and maintaining trust in a site was regular updating, demonstrated by dating information and having new information readily available (Textbox 4).

**Patients' and caregivers' quality criteria for Internet interventions relating to trustworthiness**

**Trustworthiness:**

- The site needs to be deemed trustworthy, both immediately and on subsequent or return visits.
- Trust has to be maintained, and can be lost if the site is not updated regularly.

**A good Internet Intervention will establish its trustworthiness by:**

- Being accurate
- Having no commercial links
- Not displaying advertisements
- Being authored or sponsored by a known trustworthy organization (eg, the National Health Service, a local hospital, well-known university, charity, or patient organization)
- Being regularly updated

### Respondent Validation

Of the 40 focus group participants, 37 (93% response rate) returned the postal survey ranking the criteria that emerged from the discussions. A further 20 of the 40 (response rate 50%) patients and caregivers who had consented to participate in the study but not been able to attend a focus group also returned the postal survey. Of these further 20, 8 were patients (1 had diabetes mellitus, 3 had heart disease, 2 had both heart disease and diabetes, 3 had hepatitis C) and 12 were caregivers (2 cared for people with Alzheimer's disease, 5 were parents of children with diabetes, 1 cared for both a person with Alzheimer's disease and a child with diabetes, and 4 were parents of children with asthma). Mean ratings for criteria were above 3 on a 5-point scale (from 1 = not at all important, to 5 = essential) for all but one criterion, suggesting that the analysis had identified criteria considered important by patients and caregivers. No new criteria emerged from the postal survey. The ratings and selection of the top three criteria emphasized the importance of providing useful, practical, and comprehensive information that is up-to-date, accurate, trustworthy, and easy to navigate. In line with the divergent views expressed in focus groups, many patients and caregivers rated interactive components as essential, while some rated them as unimportant.

## Discussion

### Main Findings

Participants welcomed the potential of Internet interventions but felt that many websites were not achieving their full potential. Participants generated detailed and specific criteria relating to information content, presentation, interactivity, and trustworthiness, which can be used by developers and purchasers of Internet interventions.

### Relationship With Previous Research

#### Professionally Generated Criteria

There have been a number of studies that have led to professionally generated criteria for health-related websites [11,12]. Our user-generated criteria complement and extend the professionally generated criteria, which have tended to focus on accuracy and completeness of information. Our participants expanded this focus to include control over what information is accessed, and when, as well as an emphasis on practical tips for assistance with activities of daily living. This latter type of information was not expected to be evidence based; rather, it should be based on personal experience of other users.

Moreover, although our participants' emphasis on ease of navigation is not unexpected, the emphasis on tone, visual appeal, language, and overall presentation provides practical guidance for those wishing to develop or improve a health information site.

#### Trustworthiness

Our data on how users assess trustworthiness concur with those of Eysenbach, who in an observational study of healthy volunteers in Germany found that, although users stated that the source of a website was an important feature in establishing credibility, few actually visited the "About us" section [14]. Our participants wanted instant recognition of the institution behind a site rather than taking the time to search for information about the provider. Our data extends that of Eysenbach as our participants were patients or caregivers, rather than healthy volunteers. Moreover, our data suggest that users also appraise the presence or absence of commercial sponsorship or advertisements and the frequency of updating information when considering whether to trust a site or not. This result is congruent with that from the Pew online survey, which found that strong commercial presence, out of date information, or no clear referencing of information all caused users to turn away from a site [15].

#### Interactivity and Peer Support

Our data suggest that there is considerable divergence among users about the value of interactive components and online peer support in particular. Those that were in favor of having access to online peer support, either in the form of questions and answers or online chat rooms, voiced opinions compatible with previous research in this area [19]. However, a proportion of users were opposed to online peer support, underlining that different people will want their needs met in different ways. Researchers and policy makers need to ensure that online facilities are seen as one option and must recognize that many patients or caregivers will prefer alternative facilities. Similarly, Internet interventions that contain only one interactive facility are likely to appeal to only a proportion of potential users, while those that have multiple interactive facilities are more likely to appeal to a wider range of users.

### Methodological Issues

The strengths of this study include the focus on patients and caregivers, that is, the people who are most likely to need and use Internet interventions for long-term conditions. Our sample was socioeconomically diverse and, perhaps more importantly, included a range of educational achievement and computer literacy. The use of a multidisciplinary group for analysis is known to add reflexivity and rigor [20], and we undertook a process of participant validation of results in addition to having substantial consumer input into the design, implementation, and analysis of the study. The focus group methodology allowed participants to build on each other's experiences and insights and allowed for discussion among participants to clarify ideas or concepts.

There are some limitations to this study. Participants were self-selecting volunteers who, by being motivated to participate in this kind of study, may not represent typical patients or caregivers. Although the views of caregivers and people in the United Kingdom areas other than London were represented in this sample, they were the minority. Caregivers in particular were hard to involve, and we did not have the opportunity to sample to redundancy as we did for patients. While caregivers in the study voiced similar criteria to patients, we cannot be certain that further caregiver focus groups would not have generated other criteria. The study was also limited in the extent to which participants could evaluate some of the interactive components in the 30 minutes they had with each intervention. Full appreciation of the complexities and advantages of the interactive components may require repeated use over time.

### Conclusions

Patients and caregivers welcomed the potential of Internet interventions to help users with long-term conditions take better care of their health. However, many of the currently available Internet interventions are not meeting this potential. The user-generated criteria reported in this paper should help developers and purchasers of Internet interventions provide websites that better meet users' needs.

## Multimedia Appendix 1

Foucs Group Guide.

## References

[1] Pagliari Claudia, Sloan David, Gregor Peter, Sullivan Frank, Detmer Don, Kahan James P, Oortwijn Wija, Macgillivray Steve (2005). What is eHealth (4): a scoping exercise to map the field. J Med Internet Res.

[2] Murray E, Burns J, See TJ, Lai R, Nazareth I (2005). Interactive Health Communication Applications for people with chronic disease. Cochrane Database Syst Rev.

[3] Ritterband L, Gonder-Frederick LA, Cox DJ, Clifton AD, West RW, Borowitz S (2003). Internet interventions: In review, in use, and into the future. Prof Psychol: Res Pract.

[4] Department of Health (2005). Supporting People with Long Term Conditions.

[5] Demiris George (2004). Disease management and the Internet. J Med Internet Res.

[6] Lapsley P, Groves T, The patient's journey: travelling through life with a chronic illness (2004). BMJ.

[7] Stewart DE, Wong F, Cheung AM, Dancey J, Meana M, Cameron JI, Mcandrews MP, Bunston T, Murphy J, Rosen B (2000). Information needs and decisional preferences among women with ovarian cancer. Gynecol Oncol.

[8] Cooper J (2001). Partnerships for successful self-management: The Lill Project report.

[9] Kiley Robert (2002). Does the internet harm health? Some evidence exists that the internet does harm health. BMJ.

[10] Commission of the European Communities, Communication from the Commission to the Council, the European Parliament, the Economic and Social Committee and the Committee of the Regions (2002). eEurope 2002: Quality Criteria for Health Related Websites.

[11] Eysenbach G, Powell J, Kuss O, Sa ER (2002). Empirical studies assessing the quality of health information for consumers on the world wide web: a systematic review. JAMA.

[12] Griffiths Kathleen M, Christensen Helen (2005). Website quality indicators for consumers. J Med Internet Res.

[13] Coulter A, Entwistle V, Gilbert D (1998). Informing Patients. An Assessment of the Quality of Patient Information Materials.

[14] Eysenbach Gunther, Köhler Christian (2002). How do consumers search for and appraise health information on the world wide web? Qualitative study using focus groups, usability tests, and in-depth interviews. BMJ.

[15] Fox S, Rainie L (2002). How Internet users decide what information to trust when they or their loved ones are sick.

[16] Haby MM, Waters E, Robertson CF, Gibson PG, Ducharme FM, Interventions for educating children who have attended the emergency room for asthma (2001). Cochrane Database Syst Rev.

[17] Evers KE, Cummins CO, Prochaska JO, Prochaska JM (2005). Online health behavior and disease management programs: are we ready for them? Are they ready for us?. J Med Internet Res.

[18] Richards L (2002). Using N6 in Qualitative Research. 1st edition.

[19] Eysenbach Gunther, Powell John, Englesakis Marina, Rizo Carlos, Stern Anita (2004). Health related virtual communities and electronic support groups: systematic review of the effects of online peer to peer interactions. BMJ.

[20] Barry CA, Britten N, Barber N, Bradley C, Stevenson F (1999). Using reflexivity to optimize teamwork in qualitative research. Qual Health Res.

