# Supplementary material for: Internet Interventions for Long-Term Conditions: Patient and Caregiver Quality Criteria
Source: J Med Internet Res. 2006 Jul 28;8(3):e13. doi: 10.2196/jmir.8.3.e13 (PMC1550703; doi:10.2196/jmir.8.3.e13)
Supplement: Supplementary file 1 [file jmir_v8i3e13_app1.doc]

**Multimedia Appendix**

**Focus Group Topic Guide**

Q1. Overall, what did you think about the information packages you used here?

Q2. When do you think you might use one of these?

(Probe: after the doctor had told you that you had the condition, before or after a visit to the doctor, if you were trying to make a decision about treatment, if you were explaining to a friend or relative what was wrong with you, if someone you knew thought they had a similar problem, other)

Q3. Which one did you prefer and why?

Follow up with what were the good bits in each one.

Probe group to get range of opinions and reasons for range.

Q4. Which one did you like least and why?

Follow up with what were the bad bits in each one.

Probe group to get range of opinions and reasons for range.

Q5. How do you feel about information packages like the ones you’ve just seen giving information on areas of scientific uncertainty—that is, on areas where doctors do not know the answers yet?

(Probe preferences for or against information about uncertainties about long-term effects of medical treatments, risks, and effectiveness of complementary approaches to treatment or those not yet evaluated.)

Q6. Was there anything you looked for in the information packages which you couldn’t find? If so what?

Q7. Would you buy something like this?

(Probe if so why? What would you look for? Should the NHS buy something like this? What should they look for?)

Q8. Where might you use one of these?

(Probe at home? At GP, surgery, etc. Why? Why not?)

Q9. Is there anything else you would like to say?
